# Supplementary material for: Assessing the impact of post-mortem damage and contamination on imputation performance in ancient DNA
Source: Sci Rep. 2024 Mar 14;14:6227. doi: 10.1038/s41598-024-56584-3 (PMC10940295; doi:10.1038/s41598-024-56584-3)
Supplement: Supplementary file 1 — Supplementary Figures. [file 41598_2024_56584_MOESM1_ESM.pdf]

Supplementary Information

Antonio Garrido Marques[1], Simone Rubinacci[2,3], Anna-Sapfo Malaspinas[1,4], Olivier Delaneau[5], Bárbara Sousa da Mota[1,4,*]

[1] Department of Computational Biology, University of Lausanne, Switzerland
[2] Division of Genetics, Department of Medicine, Brigham and Women's Hospital and Harvard Medical School, Boston, MA, USA
[3] Program in Medical and Population Genetics, Broad Institute of MIT and Harvard, Cambridge, MA, USA
[4] Swiss Institute of Bioinformatics, University of Lausanne, Switzerland
[5] Regeneron Genetics Center, Tarrytown, New York, USA
*Corresponding author

Supplementary Note 1: Post-mortem damage patterns

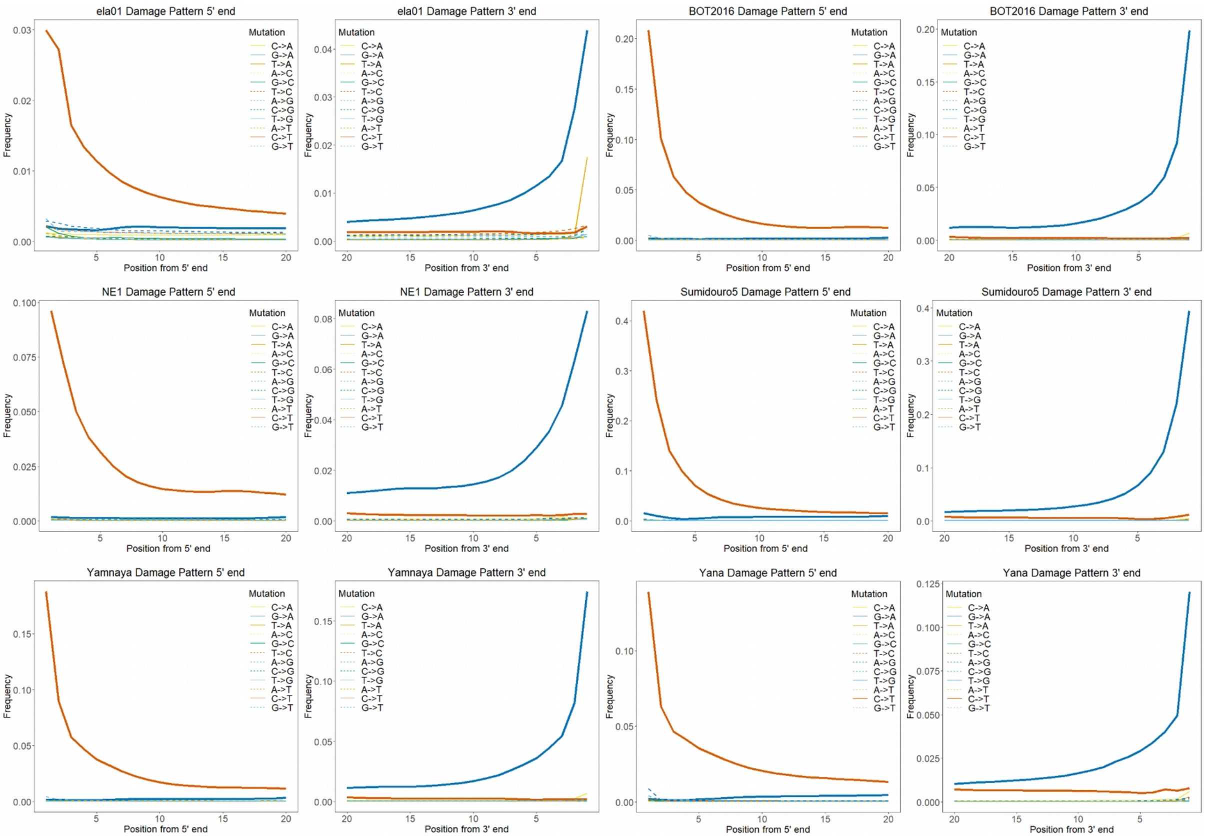

Supplementary Figure 1: Substitution rates at the ends of the reads (5' and 3' ends). Frequency of nucleotide substitutions at the 5' and 3' ends of sequencing reads measured with the bamdamage tool from bammds. Nucleotide substitution rates are presented for six of the studied ancient individual samples (ela01, BOT2016, NE1, Sumidouro5, Yamnaya, Yana). Each panel shows the substitution frequency on the *y*-axis in function of the position at the first 20 nucleotides on the *x*-axis.

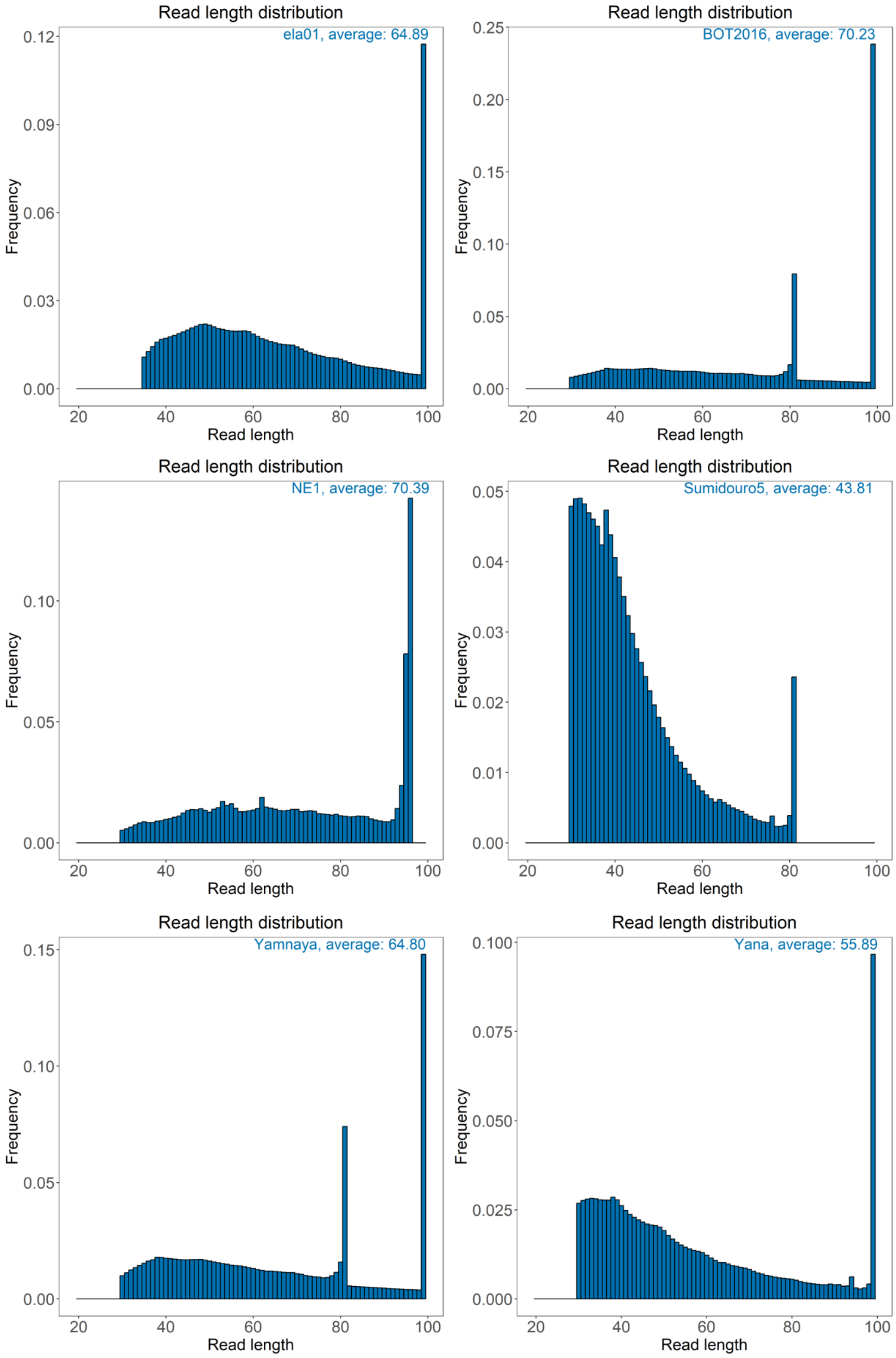

Supplementary Figure 2: Read length distribution. Distribution of the read length for six of the studied ancient individual samples (ela01, BOT2016, NE1, Sumidouro5, Yamnaya, Yana). The histograms represent the frequency of reads on the *y*-axis of a certain read length on the *x*-axis, for each sample. The average read lengths are denoted on the top right of each panel.

Supplementary Note 2: Enhanced performance of ATLAS in high-coverage genotype calling

Establishing an accurate ground truth for ancient genomes, even when high-coverage genotypes are available, is a challenge due to PMD prevalence. In this study, we found PCA to be a useful evaluation framework to compare differently generated datasets. For the high-coverage genomes, we observed that positions differently called by ATLAS and bcftools (see methods for more details on quality control procedures) display distinct spatial patterns in the PCA space (**Supplementary Figure 3**). Specifically, genotypes differently identified with ATLAS consistently positioned closer to the validation set. In contrast, those identified by bcftools were more distant, with the exception of the individual sample ela01. However, these discrepancies can be partly explained by the difference in the number of SNPs between the two as the bcftools discordant SNP set contained fewer SNPs, averaging around 10,500, resulting in an important variation in the number of SNPs across the samples when compared with the ATLAS discordant SNP set that averages approximately 472,000 SNPs (**Table S6**).

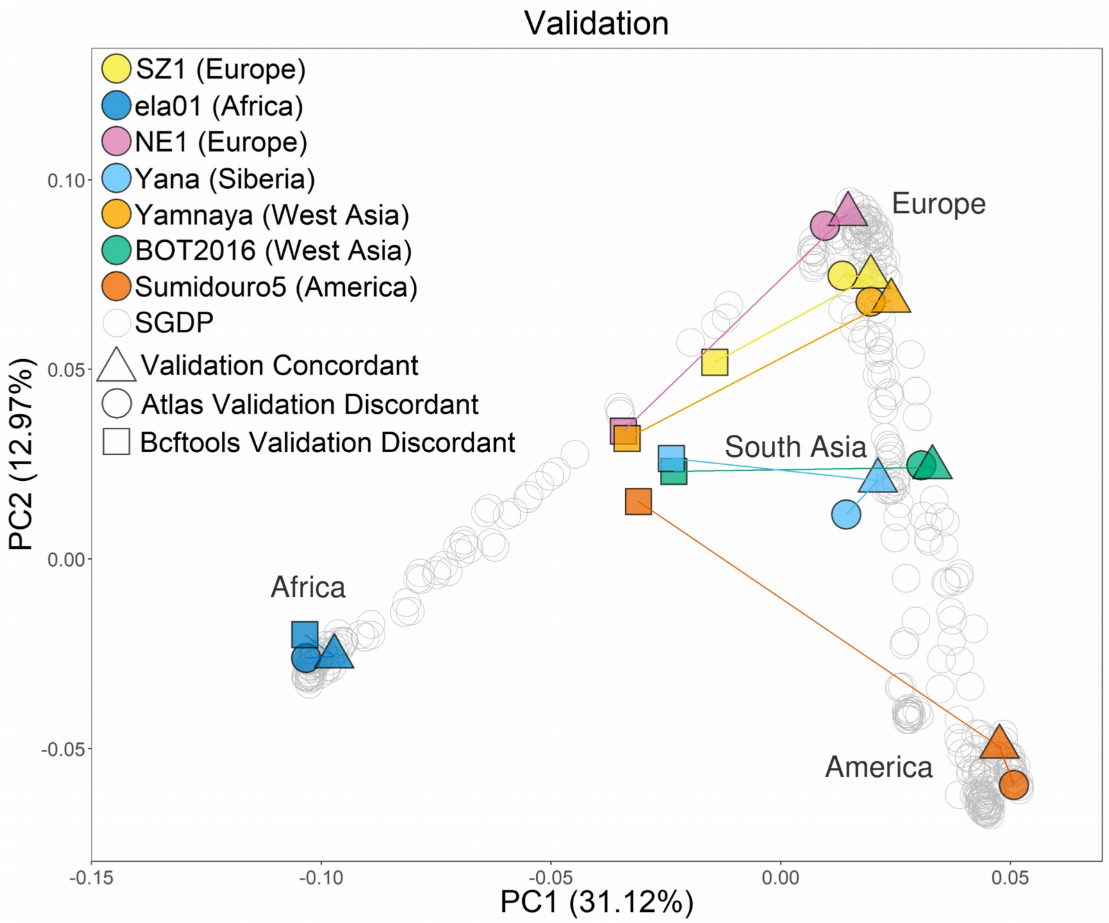

Supplementary Figure 3: PCA Validation. Two first principal components of principal component analysis (PCA) of present-day genomes (SGDP). The genetic data of seven ancient individuals using the 2.8M SNP set were projected onto it. For each individual, the triangle shape indicates the validation concordant dataset, and the circles and square shapes the positions differently identified by ATLAS (“ATLAS Imputed Discordant”) and bcftools (“Bcftools Imputed Discordant”), respectively.

Supplementary Note 3: Computational resources usage by ATLAS and bcftools to generate genotype likelihoods

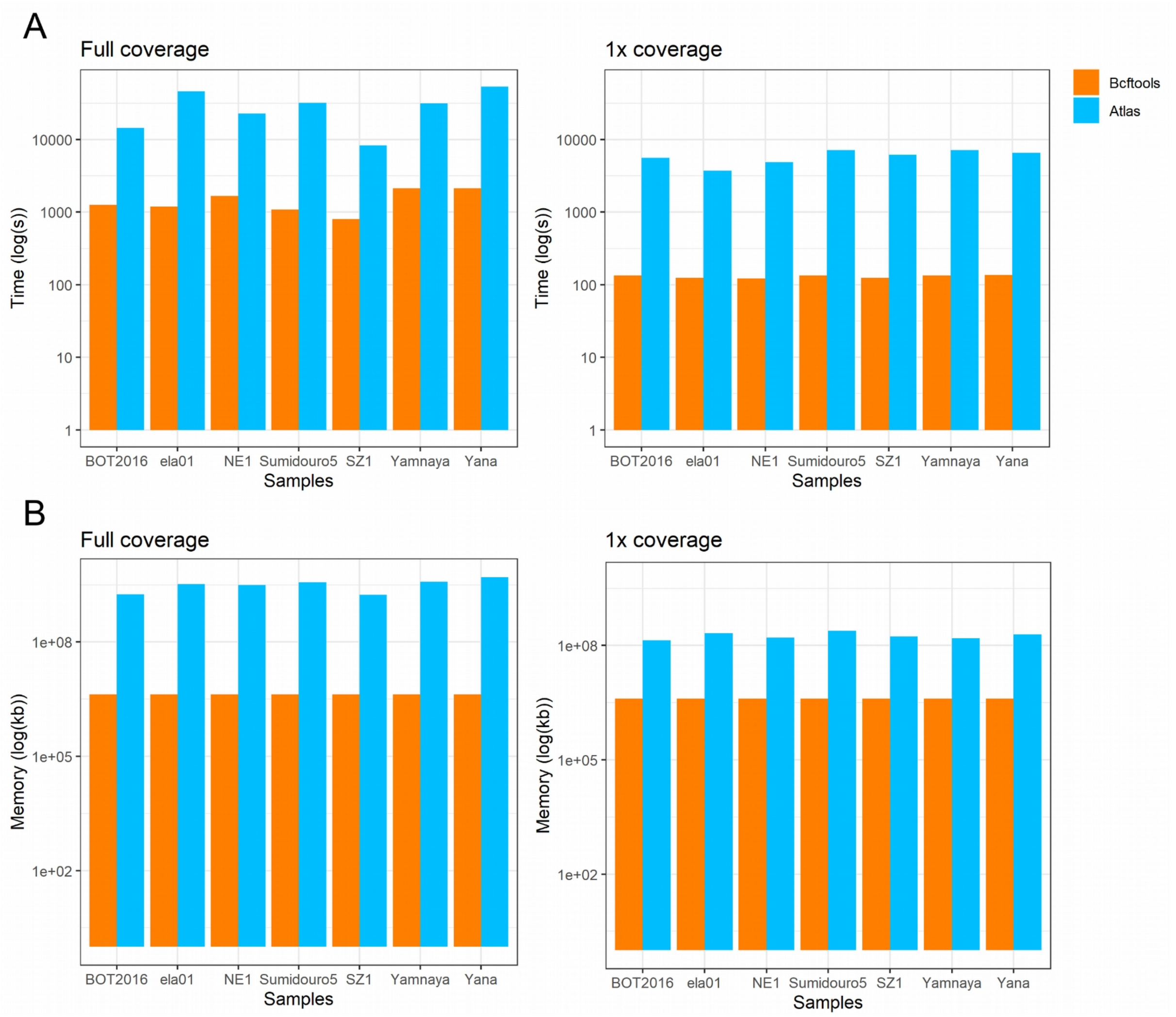

Supplementary Figure 4: Run time and computational resources when using ATLAS and bcftools. Disparity in total run time (**A**) and memory usage (**B**) between ATLAS (blue) and bcftools (orange) during the genotype calling process of seven ancient genomes. This comparison was performed on both high-coverage genomes (left) and downsampled 1x coverage genomes (right). Run time was measured on chromosome 1, while memory usage was assessed on the 22 autosomes. Results shown (*y*-axis) are represented in a log10 scale.

Supplementary Note 4: Post-mortem damage impact on imputation

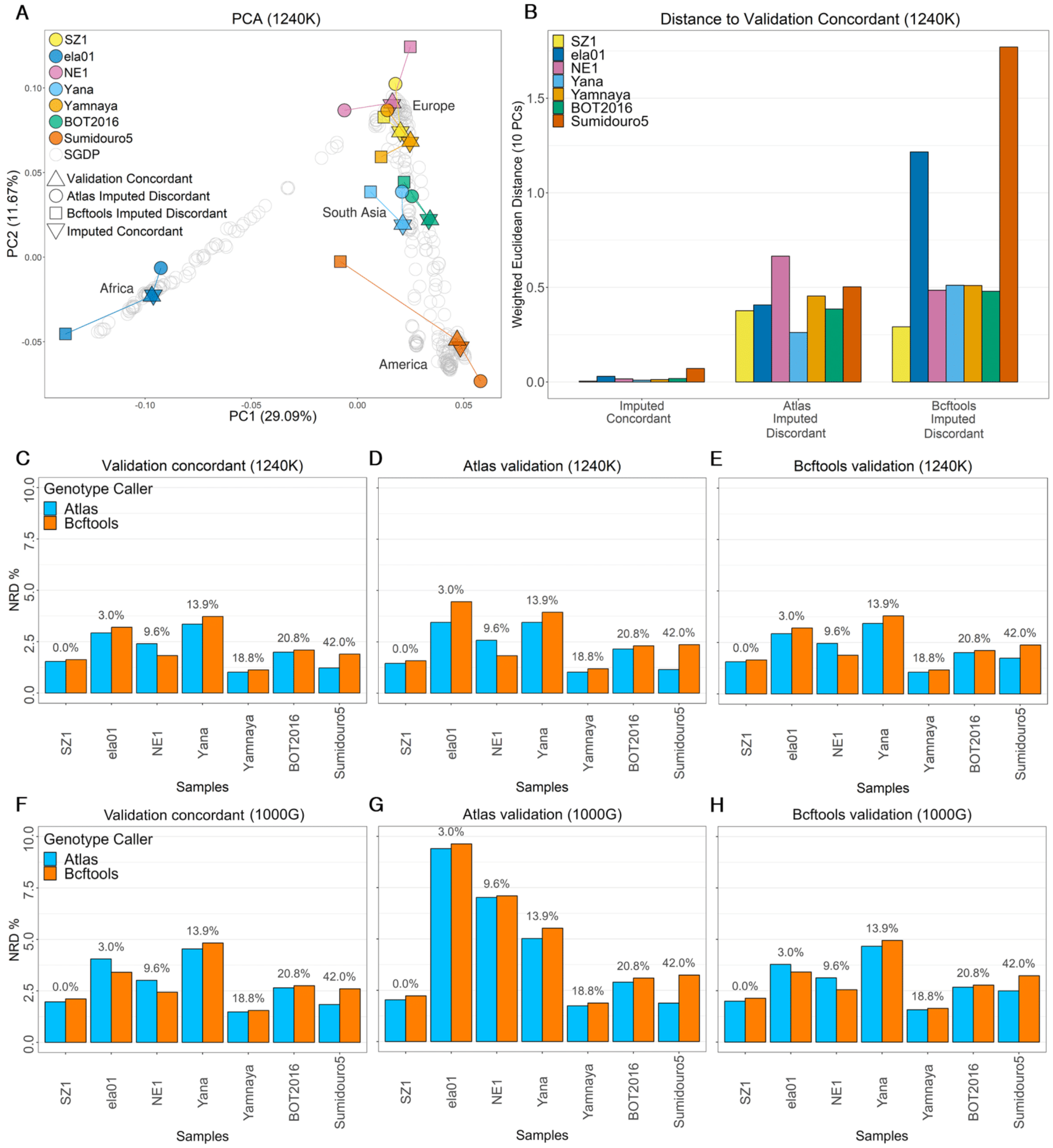

Supplementary Figure 5: Effect of two different genotype callers and SNP sets on imputation accuracy. A) Two first principal components of principal component analysis (PCA) of present-day genomes (SGDP) with the 1240k SNP set. The genetic data of seven ancient individuals were projected onto it. The triangle shape indicates the validation concordant dataset, the inverse triangle the set of imputed positions in agreement with both tools, and the circles and square shapes the positions differently identified by ATLAS ("ATLAS Imputed Discordant") and bcftools ("Bcftools Imputed Discordant"), respectively. **B**) Weighted Euclidean distances for the seven ancient individuals with the 1240k SNP set. Euclidean distances between the validation concordant set of each sample, and their concordant and discordant imputed genotypes were calculated across the first 10 PCs, with distances weighted by the eigenvalue of each PC. For additional information on the concordant and discordant samples SNP counts, refer to **Table S7. C**) Non-reference discordance (NRD) for the seven ancient individual samples with the 1240k SNP set when called with either ATLAS (blue) or bcftools (orange)

prior to imputation. The values on top of each sample indicate the PMD rate. NRD was assessed using three validation datasets: **C**) validation concordant, **D**) ATLAS validation, and **E**) bcftools validation. **F**) Assessment of NRD for the seven ancient individual samples with the 1000 Genomes (1000G) SNP set when called with either ATLAS or bcftools. The PMD rate is shown on top of each sample. The NRD was calculated using the: **F**) validation concordant, **G**) ATLAS validation, and **H**) bcftools validation datasets.

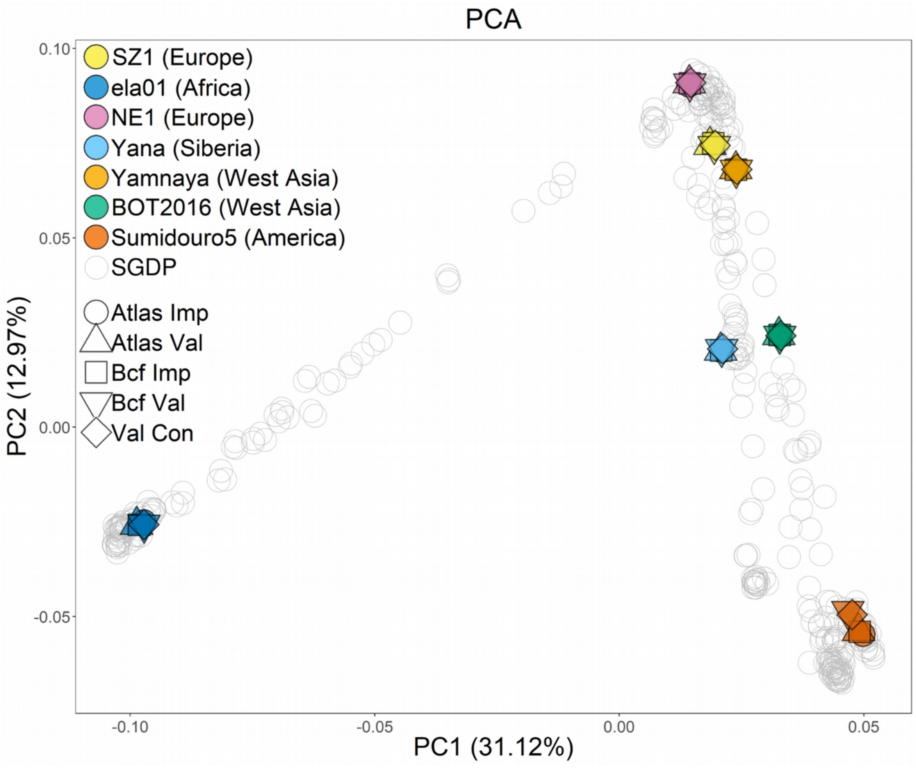

Supplementary Figure 6: Principal component analysis (PCA) of validation and imputed datasets using either ATLAS or bcftools for genotype calling. Two first principal components of PCA of present-day genomes (SGDP) with the 2.8 million SNPs set. The genetic data of seven ancient individuals were projected onto it. The triangle shape indicates the validation dataset for which SNPs were called with ATLAS ("Atlas Val"), while the inverse triangle represents validation dataset that resulted from calling genotypes with bcftools ("Bcf Val"). The intersection of these two validation datasets, that is, the "validation concordant" ("Val Con"), is represented by the diamond shape. The circle and the square indicate the imputed data for which ATLAS ("Atlas Imp") and bcftools ("Bcf Imp") were used to generate genotype likelihoods, respectively.

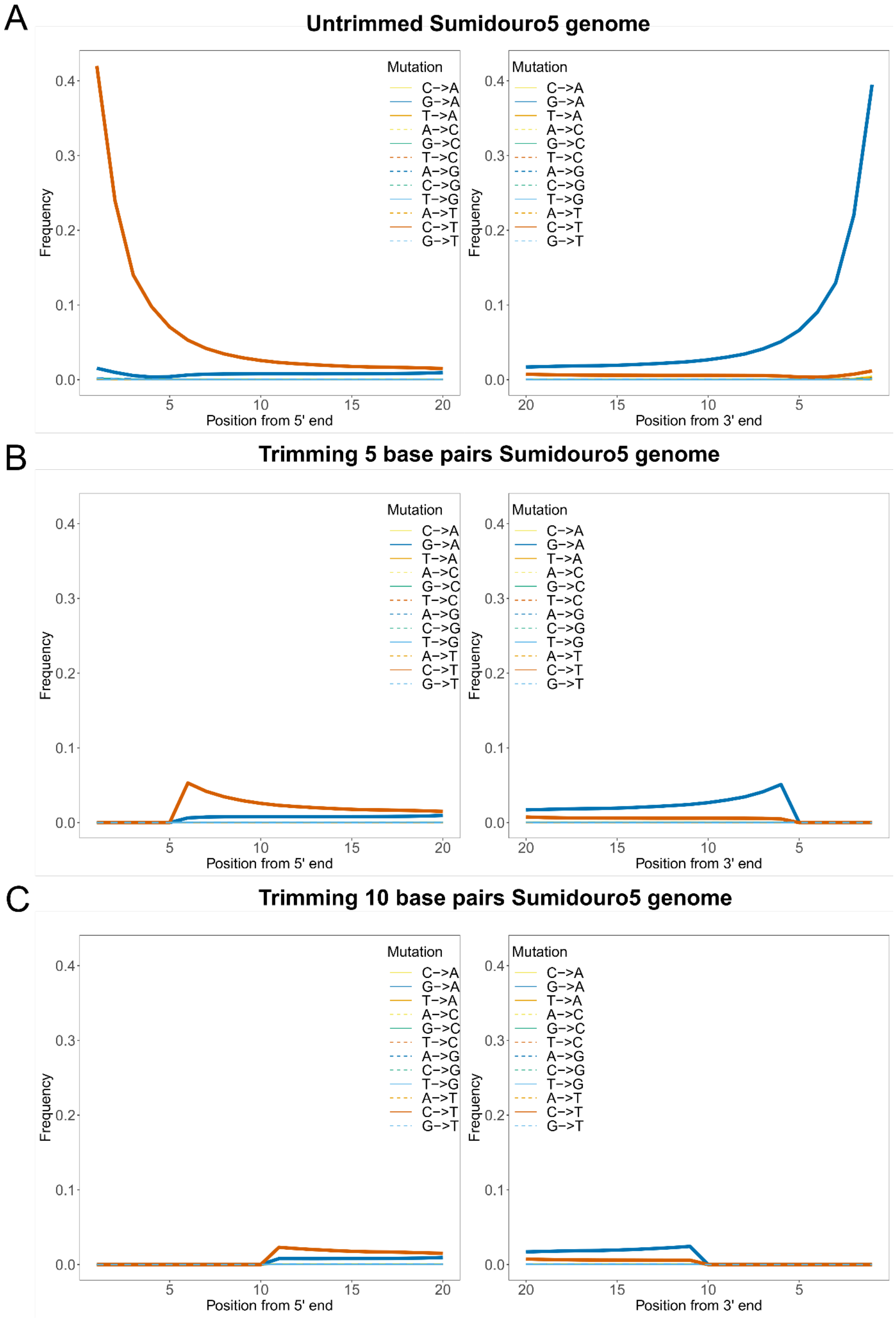

Supplementary Figure 7: Post-mortem damage (PMD) patterns for the Sumidouro5 genome with and without trimming. Frequency of miscorporations as a function of the position in the reads at the 5' end (subplots on the left) and the 3' end (subplots on the right) **A**) without trimming, when trimming **B**) five and **C**) 10 base pairs at the ends of the reads for Sumidouro5 (downsampled to 1x).

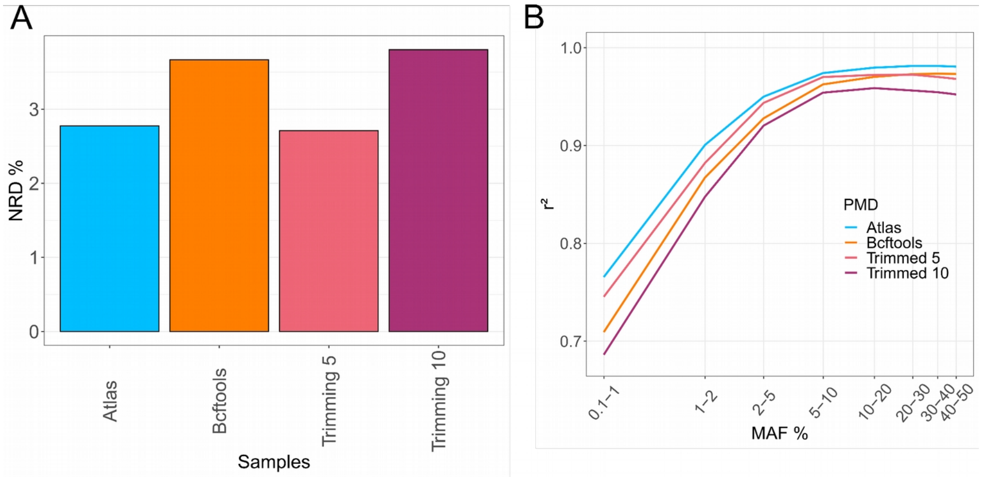

Supplementary Figure 8: Effect of trimming the ends of the reads on imputation performance of highly damaged genomes. We assessed imputation performance when using four different sets of genotype likelihoods (GLs) for imputation: i) ATLAS-generated GLs (blue), ii) bcftools-generated GLs (orange), iii) trimming of 5 base pairs at ends of the reads followed by bcftools calling (pink), and iv) trimming of 10 base pairs at ends of the reads followed by bcftools calling (dark pink). **A**) Non-reference discordance (NRD) calculated between imputed and validation genotypes (“validation concordance”). **B**) Squared Pearson correlation between imputed genotype dosages and validation genotypes (“validation concordance”) as a function of minor allele frequency (MAF).

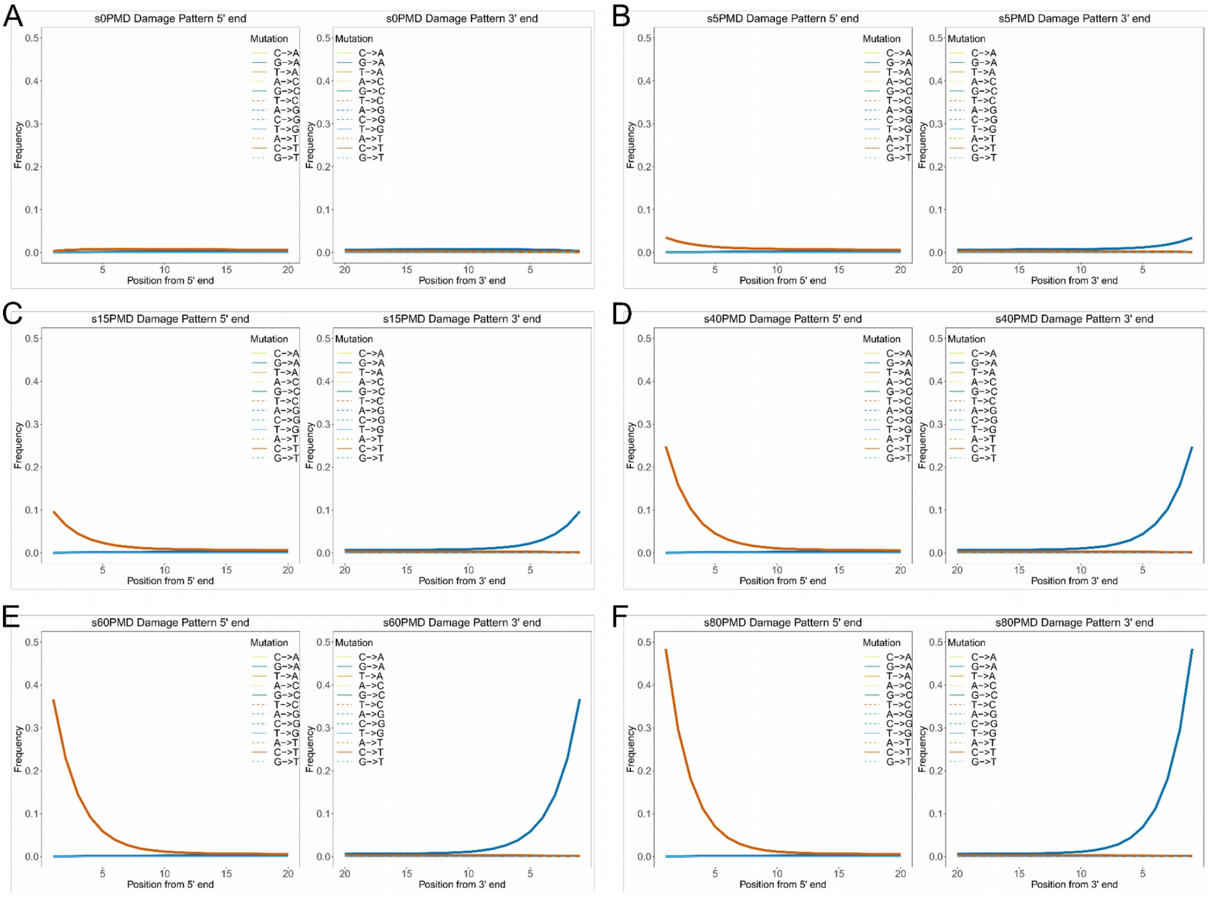

Supplementary Figure 9: Post-mortem damage (PMD) patterns for simulated ancient genomes. Frequency of miscorporations as a function of the position in the reads at the 5’ end (subplot on the left) and the 3’ end (subplot on the right) across different levels of PMD (C-to-T substitution rate at first position of the reads 5’ end) varying between 0% and 50% (A-E). The parameters we specified to run gargammel to obtain the measured PMD values are indicated in the title of the subplots (“sXPMD”).

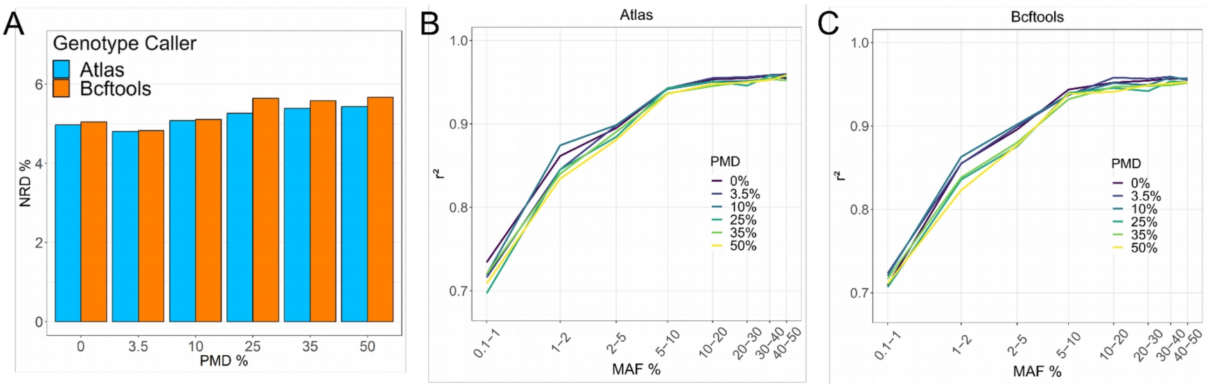

Supplementary Figure 10: Using simulated ancient genomes to evaluate the effect of PMD on imputation performance. We assessed imputation performance for simulated ancient-like 1x genomes (chromosome 1) with PMD rates varying between 0% and 50%. As validation, we used a 30x simulated genome with no PMD and using the same template as for low-coverage genomes. As before, we used two sets of genotype likelihoods (GLs): i) ATLAS-generated GLs and ii) bcftools-generated GLs. **A**) Non-reference discordance (NRD) calculated between imputed and validation genotypes across different amounts of simulated PMD and using either ATLAS or bcftools to generate GLs. We estimated squared Pearson correlation between imputed genotype dosages and validation genotypes as a function of minor allele frequency (MAF) for genomes with varying PMD and for **B**) ATLAS GLs and **C**) bcftools GLs.

Supplementary Note 5: Contamination and imputation

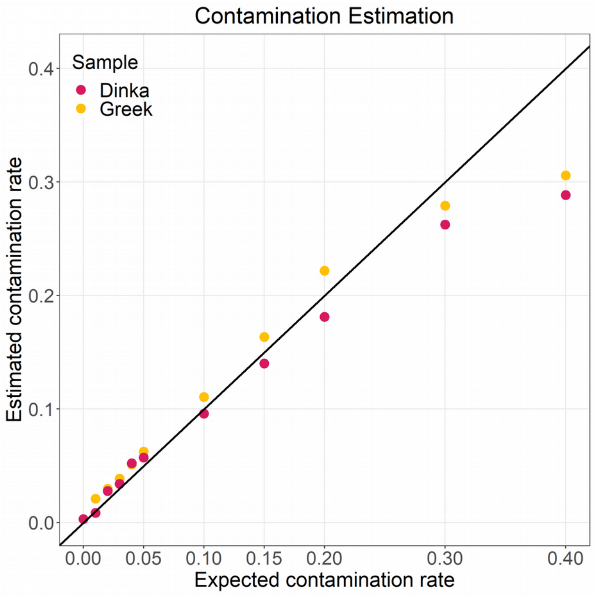

Supplementary Figure 11: Contamination estimation on chromosome X. Comparison of estimated (*y*-axis) with expected (*x*-axis) contamination rates when introducing different amounts of reads from modern human genomes in the Loschbour downsampled (1x) genome. Two contaminants were used: a present-day Greek genome in yellow, and a present-day Dinka genome in red. The black line indicates equality between expected and estimated contamination (*y*=*x*).

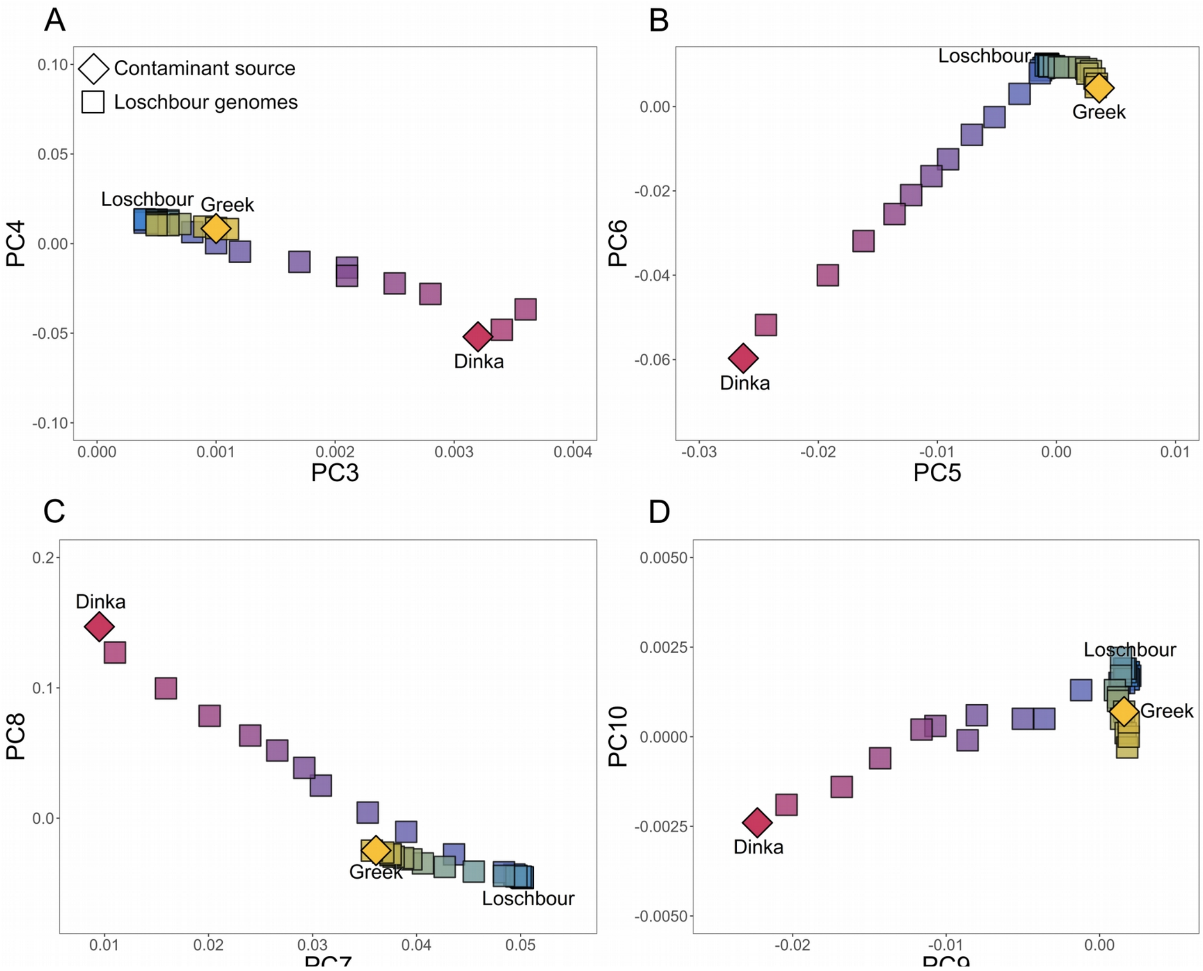

Supplementary Figure 12: Effect of contamination on genotype imputation on higher PCs. Principal component analysis (PCA) of present-day genomes, restricted to 1240k SNPs, and projection of uncontaminated and imputed contaminated ancient genomes. The ancient downsampled (1x) Loschbour genome (light blue, thicker outline) was subjected to varying degrees of contamination with DNA from a present-day Greek individual (yellow, thicker outline) and a present-day Dinka individual (red, thicker outline). All imputed contaminated downsampled Loschbour genomes are projected on top of SGDP populations (gray circles). The resultant projections are displayed across **A)** PC3 vs PC4, **B)** PC5 vs PC6, **C)** PC7 vs PC8 and **D)** PC9 vs PC10.

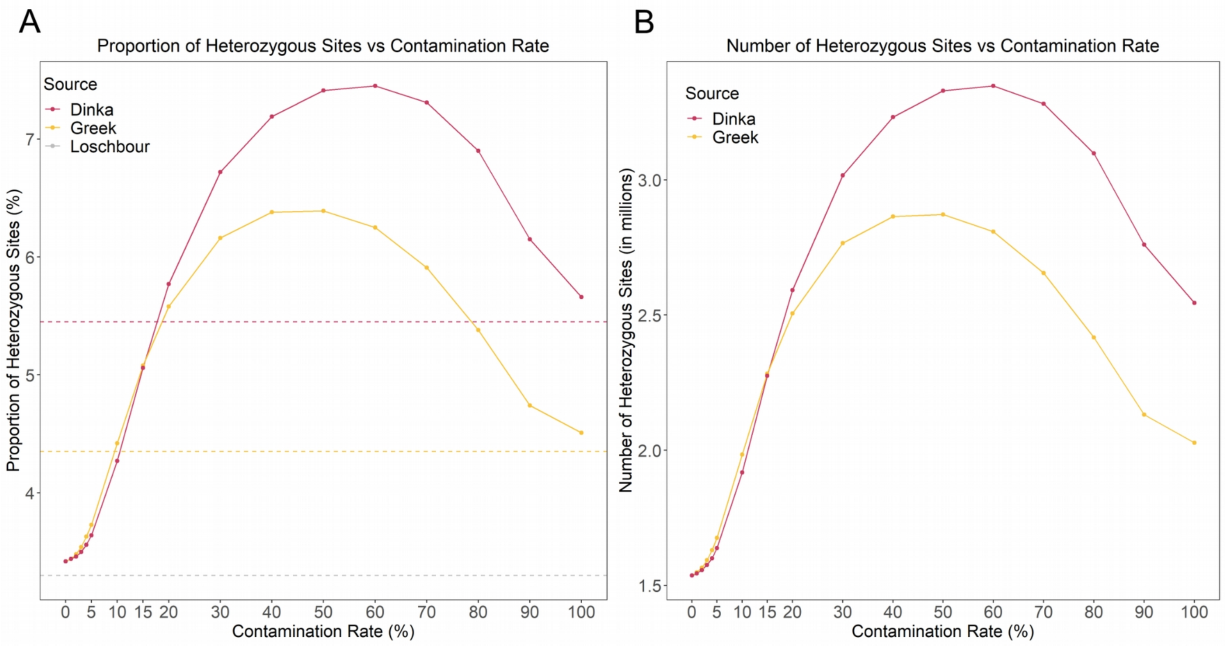

Supplementary Figure 13: Heterozygous positions across different contamination rates. Quantification of heterozygous positions across varying contamination rates ranging from 0% (imputed 1x Loschbour genome) to 100% (imputed 1x Greek or Dinka genomes) contamination. **A)** Proportion of heterozygous sites for Dinka-contaminated Loschbour genomes in red, and Greek-contaminated genomes in yellow. Dashed lines represent the proportion of heterozygous sites for the high-coverage genomes. **B)** Absolute number of heterozygous sites (in millions) for Dinka- and Greek-contaminated genomes.

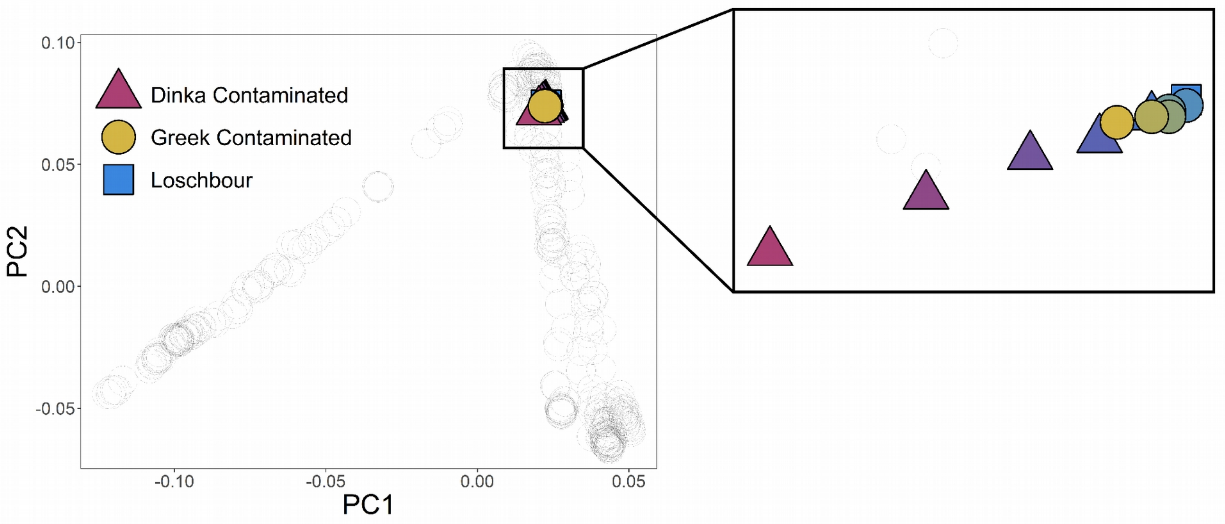

Supplementary Figure 14: Principal component analysis (PCA) focused on the effect of contamination between 0% and 5% on imputation. As before, PCA was obtained with SGDP genetic data using the 1240K sites. On the right we zoomed in on the points of interest.

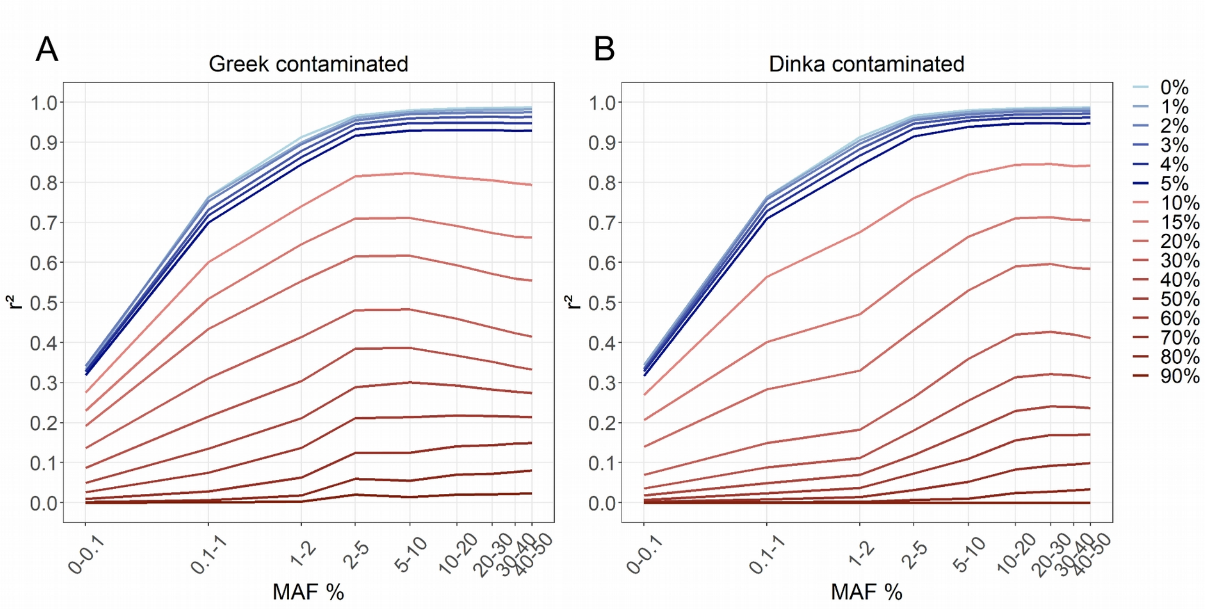

Supplementary Figure 15: Impact of contamination on genotype imputation. Mean imputation accuracy (r^2) as a function of minor allele frequency (MAF) of the downsampled contaminated Loschbour genome ranging from 0% to 90% contamination. Loschbour genome was downsampled to 1x and contaminated with **A)** present-day Greek DNA, and **B)** present-day Dinka DNA.

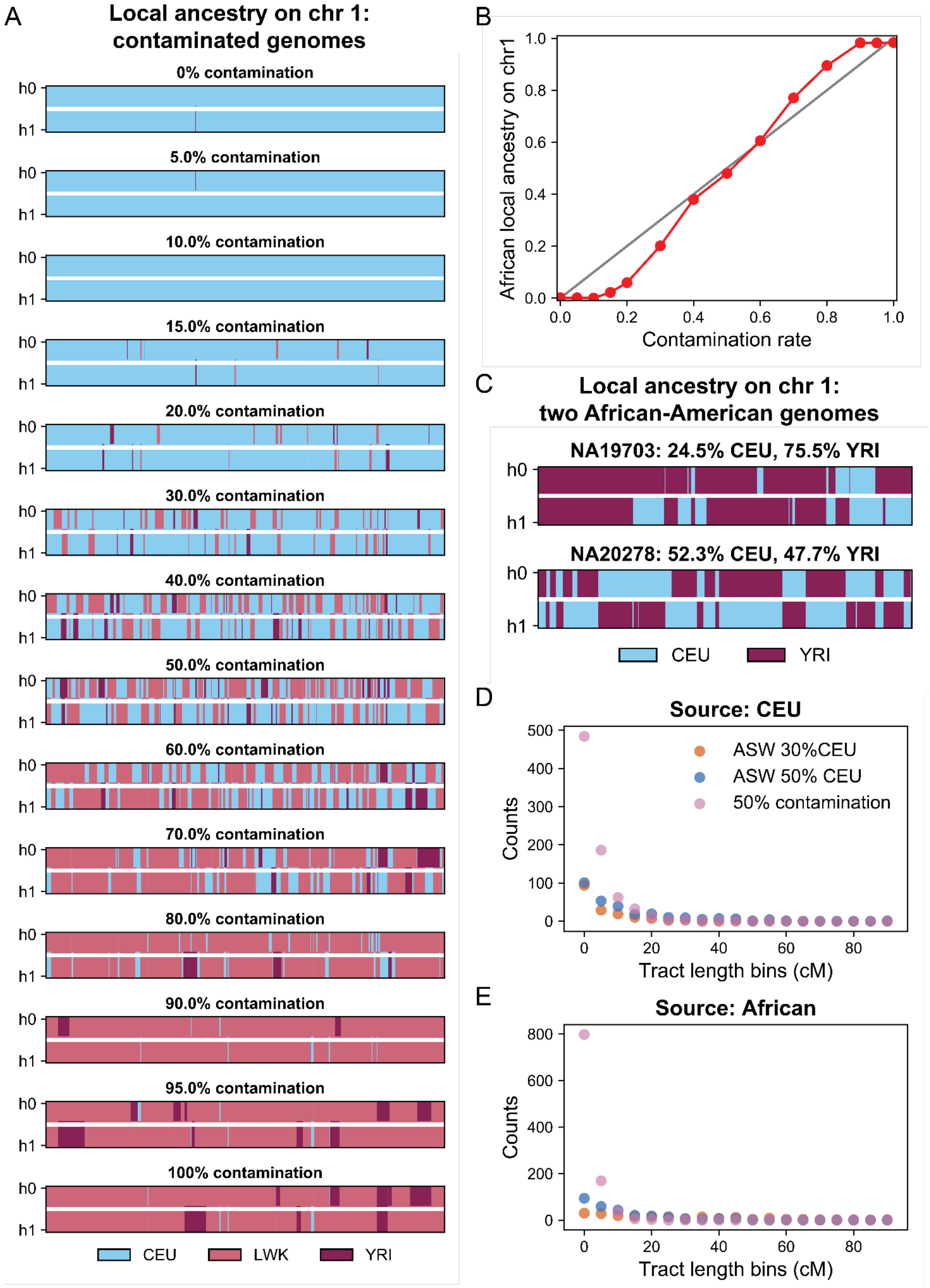

Supplementary Figure 16: Local ancestry inference for contaminated imputed chromosomes. A) Local ancestry inferred on chromosome 1 for contamination rates varying from 0% (imputed 1x Loschbour genomes) to 100% (imputed 1x Dinka genome) using three reference ancestries: CEU (Utah residents with Northern and Western European ancestry), LWK (Luhya in Webuye, Kenya) and YRI (Yoruba in Ibadan, Nigeria). **B**) Ratio of African (LWK+YRI) local ancestry on chromosome 1 as a function of contamination rate. **C**) Inferred local ancestry on chromosome 1 for two African American

individuals, NA19703 and NA20278, in the 1000 Genomes panel (ASW population label) using two reference ancestries: CEU and YRI. **D**) Distribution of CEU-inferred tracts on the African American genomes and on the contaminated Loschbour genome (50% contamination with reads from a present-day Dinka). **E**) Distribution of African-inferred tracts on the African American genomes and on the contaminated Loschbour genome (50% contamination with reads from a present-day Dinka). For the contaminated genome, African-inferred tracts include LWK and YRI tracts.

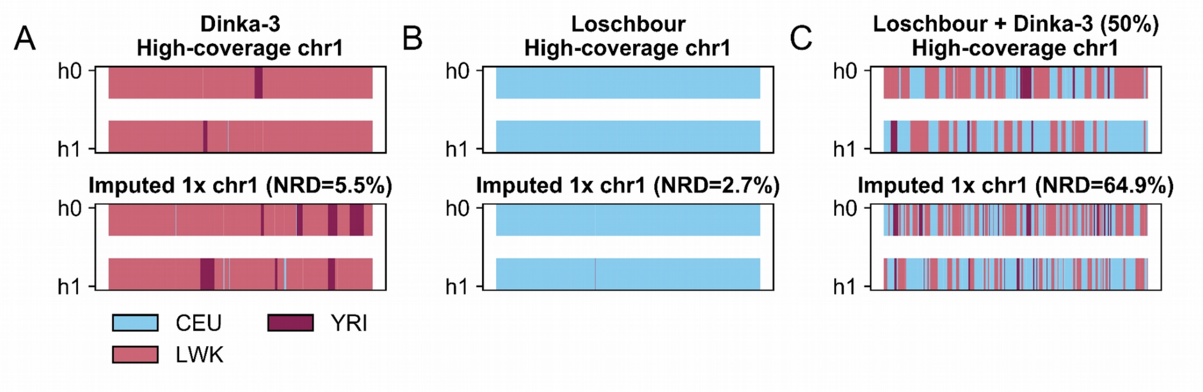

Supplementary Figure 17: Comparing local ancestry inference (LAI) in high-coverage and imputed genomes. We used three reference populations from the 1000 Genomes panel to perform LAI on chromosome 1: CEU (Utah residents with Northern and Western European ancestry), LWK (Luhya in Webuye, Kenya) and YRI (Yoruba in Ibadan, Nigeria). LAI results for high-coverage genomes are shown on top and imputed 1x genomes at the bottom: **A**) LAI results for Dinka-3 (SGDP); **B**). LAI results for Loschbour; **C**). Loschbour genome contaminated with 50% of Dinka-3 DNA. We included NRD (non-reference discordance) values for the imputed genomes. In the case of the imputed 50%-contaminated Loschbour genome, we used the original high-coverage Loschbour genome as ground truth.
